# Big food and the World Health Organization: a qualitative study of industry attempts to influence global-level non-communicable disease policy

**DOI:** 10.1136/bmjgh-2021-005216

**Published:** 2021-06-11

**Authors:** Kathrin Lauber, Harry Rutter, Anna B Gilmore

**Affiliations:** 1 Tobacco Control Research Group, Department for Health, University of Bath, Bath, UK; 2 Department of Social and Policy Sciences, University of Bath, Bath, UK

**Keywords:** health policy, nutrition, public health

## Abstract

**Introduction:**

There is an urgent need for effective action to address the over 10 million annual deaths attributable to unhealthy diets. Food industry interference with policies aimed at reducing non-communicable diseases (NCDs) is widely documented at the national level but remains under-researched at the global level. Thus, this study explores how ultra-processed food industry actors have attempted to influence NCD policy at WHO.

**Methods:**

A combination of inductive and deductive thematic coding of internal industry documents, academic literature and interviews with key informants from international organisations and global civil society was used to identify action-based strategies ultra-processed food industry actors employ to influence global-level policy.

**Results:**

Ultra-processed food industry actors have attempted to influence WHO and its policies through three main action-based strategies: coalition management, involvement in policy formulation, and information management. Coalition management includes the creation and use of overt alliances between corporations—business associations—and more covert science-focused and policy-focused intermediaries, the hiring of former WHO staff and attempted co-option of civil society organisations. Industry involvement in policy formulation is operationalised largely through the lobbying of Member States to support industry positions, and business associations gaining access to WHO through formal consultations and hearings. Information management involves funding and disseminating research favourable to commercial interests, and challenging unfavourable evidence.

**Conclusion:**

We provide novel insights into how ultra-processed food industry actors shape global-level NCD policy and identify a clear need to guard against commercial interference to advance NCD policy. In their approach, the political behaviour of multinational food corporations bears similarities to that of the tobacco industry. Increased awareness of, and safeguarding against, commercial interference at the national as well as the global level have the potential to strengthen the crucial work of WHO.

**Summary box**

**What is already known?**

- A growing body of evidence suggests that the ultra-processed food industry (UPFI) has consistently engaged in political activities to delay, weaken or prevent public health regulation, using strategies such as direct lobbying, influence through seemingly independent third parties and the production or strategic use of evidence.
- UPFI political activity at the global level remains under-researched; thus, we explore action-based strategies of UPFI actors in interaction with non-communicable disease (NCD) policy at WHO, complementing a recent investigation of arguments and framing in the same policy arena.

**What are the new findings?**

- UPFI actors have attempted to shape WHO policy on NCDs by (1) establishing and working through supportive coalitions while seeking to co-opt civil society groups, (2) harnessing or seeking access to WHO through formal and informal routes and (3) strategically disseminating favourable information.
- There are similarities with the tobacco industry’s behaviour in opposition to WHO’s efforts to advance tobacco control, although much less data are available for the food industry to date.

**What do the new findings imply?**

- Better safeguards against commercial interference and conflicts of interest at the global level as well as the national level have the potential to support WHO’s leading role in action towards better diets.

## Introduction

Unhealthy diets are a major risk factor for non-communicable diseases (NCDs) such as heart disease, type 2 diabetes and cancer.^1^ Despite ongoing calls for action on obesity and dietary NCDs, no country has successfully lowered obesity rates between 1990 and 2019.^2^ Barriers to progress include opposition of powerful commercial actors, notably the multinational ultra-processed food and beverage industry (UPFI),^3^ which we define as corporations involved in the manufacture or sale of ultra-processed foods and sugar-sweetened beverages. A growing body of evidence documents how these actors have attempted to oppose regulation at the national level, for instance, through direct and indirect lobbying of decision-makers, the creation and use of seemingly independent third parties and influence on the production and use of science.^4–7^ We use the term ‘*corporate political activity*’ to describe such behaviour. Originally developed by management scholars to describe ‘*corporate attempts to shape government policy in ways favourable to the firm*’,^8^ it has since been adopted by public health researchers using a critical lens to understand industry efforts to oppose regulation.^9 10^ In the case of the UPFI, such political activities have been identified in low-income and middle-income countries^7 11–15^ as well as high-income countries,^4 5 16–18^ but as yet, there is little empirical evidence at the global level.

In response to calls for stronger leadership from international organisations (IOs) to curb the devastating health and economic^19^ impacts of NCDs, the United Nations (UN) held their first high-level meeting (HLM) on NCDs in 2011, assembling heads of state and UN agencies to discuss action towards better prevention and treatment.^20^ This was followed by two subsequent HLMs in 2014^21^ and 2018,^22^ with a fourth scheduled for 2025. As the UN agency responsible for public health, WHO plays a key role in coordinating global efforts to prevent NCDs. Its power to set international rules which can restrict private sector activities, like the Framework Convention on Tobacco Control (FCTC),^23^ has been described as ‘*political dynamite*’.^24^ Although WHO has published technical guidance on obesity and dietary NCDs, Member State-led political decisions remain unambitious (key developments in table 1).^25^ Public health advocates largely greeted the political declaration of the third HLM^22^ and what were intended as ‘*bold recommendations*’ from the WHO independent high-level commission^26^ to the HLM with disappointment. The declaration was criticised for its weak language on NCD interventions and financing^27 28^ and the commission’s report for producing recommendations weaker than existing WHO guidance after a US representative ‘*torpedoed*’^29^ efforts to include a recommendation to tax sugar-sweetened beverages.^30 31^ UPFI representatives, on the other hand, welcomed both documents, lauding calls for public-private collaboration in particular.^29 32^

**Table 1:** Key WHO/UN publications and events relating to obesity and dietary NCDs from 2000 onwards

Year	Title	Detail
2000	Global strategy for the prevention and control of NCDs^135^	The strategy was adopted at the 53rd World Health Assembly (WHA).
2003	Diet, nutrition and the prevention of chronic diseases (TRS 916)^136^	Report of the joint WHO/FAO expert consultation with Member States, UN agencies, civil society and the private sector.^137^ It includes a recommendation to limit free sugar intake to 10% of calorie intake.
2004	Global strategy on diet, physical activity and health^138^	The strategy was mandated by Member States at the 55th and endorsed at the 57th WHA. After opposition from industry and some Member States to *TRS 916*, reference to the expert report and its key recommendations was dropped from the final strategy.
2008	2008–2013 action plan for the global strategy for the prevention and control of NCDs^138^	The action plan draws on the 2000 global strategy and the 2004 global strategy, setting out six key objectives. It was endorsed at the 61st WHA.
2010	Set of recommendations on the marketing of foods and non-alcoholic beverages to children^139^	The set of evidence-based recommendations was endorsed at the 62nd WHA and followed up by a mandate to develop technical guidance to support the implementation of the recommendations.
2011	WHO global forum: addressing the challenge of NCDs^140^	In the lead-up to the 2011 HLM, the global forum was held in Moscow as a multistakeholder forum which brought together Member States and a range of non-state actors, including the private sector.
2011	*First UN HLM on NCDs:* political declaration of the first HLM on NCDs^20^	The political declaration of the first HLM on NCDs was adopted at the 66th UN General Assembly.
2012	A framework for implementing the set of recommendations on the marketing of foods and non-alcoholic beverages to children^141^	Technical guidance on the implementation of the set of recommendations was provided following a mandate by the 62nd WHA.
2013	Global action plan for the prevention and control of NCDs 2013–2020^1^	The 2013–2020 action plan, replacing the 2008–2013 action plan, was adopted at the 66th WHA. It sets nine voluntary targets, including a 25% reduction in premature mortality from NCDs by 2025, and provides policy recommendations to achieve these.
2014	Rome declaration on nutrition (and framework for action)^142 143^	Outcome documents of the Second International Conference on Nutrition (ICN2), which was convened jointly by WHO and FAO, and attended by ‘*nearly 100 (representatives) from the business community’*.^144^
2014	*Second UN HLM on NCDs:* outcome document of the second HLM on NCDs^21^	The second HLM took place to review and assess progress towards NCD targets. The outcome document was adopted at the 68th UN General Assembly.
2015	Guideline: sugars intake for adults and children^65^	Part of the effort to reach targets set by the 2013–2020 action plan and based on a review of the scientific evidence and expert consultation, the new guidelines recommend a reduction of daily intake of free sugars to <10% of total energy intake, with a reduction to below 5% recommended for further health benefits.
2016	Sustainable Development Goals (SDGs)^145^	Ratified in 2015, the 17 SDGs replaced the Millennium Development Goals on 1 January 2016. SDG 3 is focused on health, but many others relate to food and nutrition.^146^
2016	Fiscal policies for diet and the prevention of NCDs^147^	Based on a technical meeting held on 5–6 May 2015 in response to the increasing number of Member State requesting guidance on fiscal policies for health. The report supports sugar-sweetened beverage taxes.
2016	Report of the commission on ending childhood obesity^148^	The commission was established in 2014 by the Director General, and its final report welcomed at the 69th WHA in 2016. The process leading up to the report included consultation with the private sector and civil society.
2017	Montevideo roadmap 2018–2030 on NCDs as a sustainable development priority^149^	The roadmap is the outcome document of the WHO global conference on NCDs, a high-level event. The process leading up to the conference included a public consultation.
2017	Tackling NCDs: ‘best buys’ and other recommended interventions for the prevention and control of NCDs^150^	Appendix 3 of the 2013–2020 action plan was endorsed at the 70th WHA with resolution WHA70.11. It identifies a menu of policy options for Member States, part of which is the set of cost-effective *best buys*. Italy and the USA did not endorse the updated set of *best buys and other recommended interventions*, dissociating themselves from the relevant paragraph of WHA70.11.^151^
2018	Time to deliver: report of the WHO independent high-level commission on NCDs^26^	The first report of the high-level commission on NCDs, tasked with advising the WHO Director General on accelerating progress against NCDs, was published following a public consultation and provided six recommendations. It was welcomed at the 73rd UN General Assembly.
2018	*Third UN HLM on NCDs*: political declaration of the third HLM on NCDs^22^	Adopted at the 73rd UN General Assembly, following the third HLM on NCDs, the political declaration reaffirms commitments to address NCDs globally and schedules the next HLM for 2025.
2019	Final report of the WHO independent high-level commission on NCDs: it is time to walk the talk^152^	The second and final report of the WHO independent high-level commission on NCDs delivers a number of recommendations to WHO, including scaling up private sector engagement.

FAO, Food and Agriculture Organization; HLM, high-level meeting; NCD, non-communicable disease; TRS, technical report series; UN, United Nations.

Another point of contention is WHO’s contrasting approach to the unhealthy commodity industries which drive NCDs and have a history of impeding policy progress at national level.^33^ The agency has a strict policy of non-engagement with the tobacco industry as mandated by Article 5.3 of the FCTC,^23^ but its Framework of Engagement with Non-State Actors (FENSA)^34^ has been described as an open door rather than a fence for other commercial actors, including the UPFI.^35^ The new *official relations* status—which business associations, philanthropic foundations and non-governmental organisations (NGOs) can apply for—is not a requirement for WHO to engage with a non-state actor but provides privileged access, for instance, allowing participation in WHO governing bodies meetings.^36^ Welcomed by the private sector when it was adopted in 2016 after 4 years of negotiation with Member States, FENSA was again deemed insufficient by public health advocates.^37^

In light of the lack of progress in addressing a major cause of ill health globally, there is an urgent need to better understand how the UPFI attempts to influence global-level NCD policy.^38^ We aim to address this gap by harnessing conceptual developments from tobacco control research, which were significantly advanced by the release of millions of internal tobacco industry documents through litigation in the 1990s.^39^ These documents offer unique and detailed insights into corporate policy influencing strategies in a way which has not hitherto been possible for the UPFI: in January 2021, the Tobacco Industry Documents Archive^40^ hosted 12 220 370 documents, whereas its food industry counterpart only contained 131 865.^41^ An evidence-based model of tobacco industry political activity, the Policy Dystopia Model (PDM)^10^ has been developed using two systematic reviews of tobacco industry document research. It categorises corporate political activity into argument-based and action-based strategies, showing how they work collectively to construct and disseminate a narrative that regulatory policies will fail and lead to undesirable consequences. The PDM presents two hierarchical taxonomies of instrumental (action-based) and discursive (argument-based) strategies with the respective subcategories of techniques and arguments. Researchers have successfully applied the PDM to study political activities of the UPFI and other industries at the national level, demonstrating its applicability beyond tobacco control.^13 14 42–44^ Our study harnesses the PDM to examine UPFI instrumental strategies at the global level. In doing so, it builds on our recent work which examined the UPFI’s discursive strategies in WHO consultations.^45^ The study aims to:

- examine UPFI political activity—specifically its instrumental strategies—aimed at WHO.

Additionally, we seek to explore how this differs with the better documented tobacco industry political activities against WHO’s public health efforts,^46–48^ and how public health actors explain these differences.

## Methods

We drew on multiple sources to map the instrumental strategies UPFI actors use, focusing on post-2000 policy developments around obesity and dietary NCDs at WHO headquarters. UPFI actors were defined as including corporations involved in the manufacture of ultra-processed products, forming a key part of their supply chain, or holding a financial interest in the sale of these products, including ingredient supply and processing, fast food restaurant organisations, and retail.^45^

### Data

We triangulated key informant interviews with a review of academic literature and UPFI documents published via the Food Industry Documents Archive^41^ to gain a more comprehensive understanding.^49^

#### Interviews

We conducted semi-structured interviews with 16 key informants who had 3 or more years of experience in supranational NCD policymaking and had closely participated in WHO processes. Interviewees were identified through purposive and snowball sampling and approached via email. The interview schedule—developed based on authors’ knowledge of the topic, reading of key literature, and the PDM—used open questions and focused probing, allowing for exploration of topics interviewees perceived as particularly relevant.^50 51^ The lead author conducted interviews remotely (n=13) or in person (n=2) between October 2019 and June 2020. Four informants were current employees of an IO, 2 were former IO employees and 10 were advocates who engaged with WHO on NCD policy (table 2). Two participants were interviewed together (CS-2). Interviews lasted between 27 and 101 min, averaging approximately an hour. If participants agreed to be recorded, the lead author transcribed the interviews. Two interviews were not audio-recorded on the participants’ request, instead detailed notes were taken and approved. All interviewees provided informed consent and were fully anonymised due to the sensitive nature of the topic.

**Table 2:** Interviews: all participants are identified through their primary role

Interviewee code	Background of interviewee(s)	Interview mode and duration
IO-1	Current IO employee with NCD-relevant remit	In person, 51 min
IO-2	Current IO employee with NCD-relevant remit	Remotely, 44 min
IO-3	Current IO employee with NCD-relevant remit	Remotely, 49 min
IO-4	Current IO employee with NCD-relevant remit	Remotely, 46 min
Ex-IO-1	Former IO employee/CS member	Remotely, 47 min
Ex-IO-2	Former IO employee/academic	Remotely, 1 hour 11 min
CS-1	CS member, previously IO	Remotely, 1 hour 3 min
CS-2	Joint interview of two CS members	Remotely, not recorded
CS-3	CS member/academic	Remotely, not recorded
CS-4	CS member	Remotely, 27 min
CS-5	CS member	Remotely, 28 min
CS-6	CS member	Remotely, 1 hour 41 min
CS-7	CS member	In person, 1 hour 17 min
CS-8	CS member	Remotely, 46 min
CS-9	CS member	Remotely, 50 min

CS, civil society; IO, international organisation; NCD, non-communicable disease.

#### Literature and document searches

We conducted systematic searches in Web of Science, PubMed, Scopus, and Google Scholar using combinations of the following terms: food/beverage/sugar, corporate/commercial/industry, political activity/influence/interference/involvement, lobby*, ‘world health organization’, ‘united nations’ (details in online supplemental file 1). We included pieces which were published in peer-reviewed journals after 1999 and contained concrete information on political activities (rather than arguments or market strategies) of UPFI actors described as targeting WHO in relation to NCDs. Additional literature, including source material cited and providing evidence of UPFI conduct, was identified from bibliographies of included studies. The review was initially conducted in September 2019 and updated in October 2020, identifying 21 articles, of which only 10 presented primary research (online supplemental file 2).

10.1136/bmjgh-2021-005216.supp1 Supplementary data

10.1136/bmjgh-2021-005216.supp2 Supplementary data

We searched the Food Industry Documents Archive using combinations of the terms ‘world health organization’, ‘united nations’ and ‘world health assembly’. Documents were included if they contained information on actual or planned UPFI activities in relation to WHO after 2000. Searches identified 16 documents which had been released through leaks, litigation or freedom of information requests (online supplemental file 2).

Targeted follow-up web searches (Google, WHO, key UPFI/third-party actor websites) were conducted to corroborate and expand on information identified from interviews, literature or documents. We only name corporations where we have supporting documentary evidence.

### Analysis

We adopted the strategies from the PDM to structure our analysis but identified techniques inductively to ensure novel insights from the global-level context are captured. When analysing UPFI documents, we followed a hermeneutic approach, starting by reading and re-reading them and carefully considering their meaning and the context in which they were produced throughout the analysis.^52^ During an initial reading of all data, we confirmed that the PDM was an appropriate analytical framework and made the decision that no additional strategies needed to be created.

The lead author coded interview data, internal documents, and literature in three main steps, using a combination of inductive and deductive thematic analysis^53^ with a latent coding approach to create a hierarchical framework of UPFI behaviour. First, individual industry actions were identified inductively as the smallest unit of analysis. They were judged relevant if they fit conceptually into one of the instrumental strategies from the PDM. Second, they were grouped into techniques based on conceptual coherence. To be recorded as such, a technique had to be supported by at least two data points and verified by documentary evidence where possible. Third, we grouped techniques into instrumental strategies from the PDM. The framework developed by the lead author was refined iteratively in discussion with the wider research team.

Analyses were conducted using NVivo V.12.^54^

## Results

We identified numerous actions which could be collapsed into 10 techniques and 3 overarching instrumental strategies which interlink and reinforce each other: coalition management, UPFI involvement and influence in policy, and information management (table 3). In this section, we present the hierarchical framework which resulted from our analysis. While we present our results through a global lens, it was clear that some attempts at influence were operationalised through the national level.

**Table 3:** UPFI instrumental strategies and techniques with substantiating evidence from interviews and document/literature searches^10^

Strategy	Definition	Technique	Supportive evidence		
			Interviews	Internal documents	Literature
Coalition management	Building or managing alliances with other corporations or societal actors to establish alternative platforms for arguments or routes for access	Harnessing access to Member States	IO-1, IO-2, IO-3, IO-4, ex-IO-1, ex-IO-2, CS-2, CS-3, CS-4, CS-5, CS-6, CS-7, CS-8, CS-9	55–57 153 154	58 59 72 155–157
		Engaging in business coalitions	IO-2, IO-4, ex-IO-2, CS-1, CS-4, CS-6, CS-7, CS-9	57 60 154	56 58 59 72 74 89 105
		Working through science/policy intermediaries	IO-3, ex-IO-1, ex-IO-2, CS-1, CS-2, CS-7, CS-8	57 78 101 102 158	17 56 58 59 79 80 84 89 159
		Co-opting civil society	IO-3, ex-IO-1, ex-IO-2, CS-5, CS-6, CS-7, CS-8, CS-9	–	38 89
		Hiring former WHO staff	CS-1	–	89
Involvement and influence in policymaking	Gaining or maintaining access to, and seeking representation or involvement in policymaking, including direct lobbying of policymakers	Participating in WHO processes	IO-1, IO-2, IO-3, IO-4, ex-IO-1, ex-IO-2, CS-1CS-4, CS-5, CS-6, CS-8	60	38 56 58 59 74 75 89
		Intimidating policymakers	Ex-IO-1, ex-IO-2, IO-4	–	–
Information management	Sponsoring, producing or disseminating favourable information while suppressing and undermining unfavourable information	Sponsoring or disseminating favourable information	IO-1, ex-IO-1, ex-IO-2, CS-1, CS-2	60	56 59 80 89 159
		Challenging or undermining unfavourable information	IO-1, CS-5	60	56 58 59 74 79 80
		Managing own image/engaging in corporate social responsibility activities	IO-1, IO-2, CS-6, CS-7	106	89 105 107

CS, civil society; IO, international organisation; UPFI, ultra-processed food industry.

### Coalition management

Our analysis suggests that the UPFI created and used multiple voices and alliances to support its positions and gain routes for access, while attempting to weaken potential opponents through co-option.

#### Harnessing access to WHO Members States

WHO Member States—most frequently countries in which multinational food corporations are headquartered—were highlighted as a crucial path for UPFI influence by nearly all interviewees, in internal documents and the literature. One IO employee noted: *‘my experience is always that [food corporations] work through Member States, they influence, and they weaken the language. That’s more […], at country level, where they directly lobby*’ (IO-4). Evidence indicates that UPFI actors actively lobbied national government bodies to promote UPFI-favourable positions in WHO negotiations (IO-4, ex-IO-2, CS-4, CS-5, CS-6, CS-8, CS-9).^55–61^ In this context, 3 interviewees reported that the risk of influence on political documents, agreed by Member State consensus, was greater than on technical guidance based on evidence review and expert advisory groups (IO-1, ex-IO-1, CS-2). Such involvement was described as a relatively recent phenomenon: 1 interviewee with two decades of professional experience reflected: ‘*NCDs have really emerged over the last six years, seven years, […]. That’s where [the UPFI] started to penetrate the discourse. So, [food company] didn’t really have much interest in the neglected diseases or infectious diseases arena’* (CS-8).

Interviewees highlighted 2 Member States as particularly aligned with commercial interests: Italy and the USA. Italy has opposed guidelines for sugar and meat reduction, and front-of-pack labelling policies^62 63^—a stance often positioned as defending the Mediterranean diet^64^ but attributed by participants to the political power the confectionery manufacturer Ferrero and other UPFI actors hold in Italy (CS-1, ex-IO-1, IO-3, IO-4). For example, Italy strongly opposed WHO’s 2015 sugar guidelines,^65^ which included a recommendation to decrease consumption to 5% or less of total calorie intake,^66^ and at the 136th WHO Executive Board meeting leading up to the publication of the guidelines called for an urgent review of WHO’s technical guideline development process towards greater involvement of Member States and ‘*other stakeholders’*.^67^ The Italian delegation to this meeting included an ‘*expert*’ to the Ministry of Foreign Affairs who had appeared as a senior advisor to Ferrero until shortly before (CS-3, CS-8).^66 68 69^ Notably, Italy protested against the participation of a WHO official in the EAT-Lancet Commission on Food, Planet and Health, and WHO’s decision to host the launch event,^64 70^ although we obtained no evidence of UPFI involvement in this.

The USA was a key player in the possibly best-documented case of UPFI interference with WHO policymaking. The Sugar Association and other UPFI groups mounted a concerted campaign against the 2004 Global Strategy on Diet, Physical Activity and Health, and the science underlying it. UPFI efforts focused particularly on the recommendation to restrict added sugar intake to 10% of daily energy consumption. The Sugar Association wrote to the then US Minister of Health, asking him to withdraw US funding to WHO unless the recommendation was removed.^71^ This prompted a US official to oppose the recommendation in a letter to WHO.^56 59 72 73^ Interviewees noted that the USA had continued to oppose regulatory approaches in favour of voluntary or partnership measures (IO-1, IO-2, IO-4, CS-1, CS-5, CS-6)^74 75^ and appeared aligned with UPFI interests (CS-1, CS-2, CS-5, CS-6, CS-7, CS-8, CS-9). One participant contended that a statement from the US Chamber of Commerce opposing the Trump administration’s 2020 move to end its WHO membership^76^ signalled the industry’s concern that ‘*without their friends in the US government trying to call the shots at WHO, they have less ability to influence the agenda at WHO’* (CS-9).

#### Business coalitions

Formal WHO interactions with the UPFI primarily take place through business associations,^45^ in line with FENSA and WHO’s preference to avoid association with one individual company over another (IO-2).^34^ A set of UPFI-specific and multi-industry business associations specialising in interacting with the UN system is discernible,^45^ and efforts in the obesity space are spearheaded by the International Food and Beverage Alliance (IFBA, box 1), which unites 12 food and beverage multinationals. UPFI corporations tend to be represented in multiple business associations which coordinate efforts and provide numerous routes to global policymakers.^45^ For instance, a delegation of US Council for International Business members, including Ferrero, met with WHO and other IOs in 2018 ‘*to highlight American policy priorities and concerns’*.^77^

Box 1 **The International Food and Beverage Alliance (IFBA)**

**What is already known?**

- A growing body of evidence suggests that the ultra-processed food industry (UPFI) has consistently engaged in political activities to delay, weaken or prevent public health regulation, using strategies such as direct lobbying, influence through seemingly independent third parties and the production or strategic use of evidence.
- UPFI political activity at the global level remains under-researched; thus, we explore action-based strategies of UPFI actors in interaction with non-communicable disease (NCD) policy at WHO, complementing a recent investigation of arguments and framing in the same policy arena.

**What are the new findings?**

- UPFI actors have attempted to shape WHO policy on NCDs by (1) establishing and working through supportive coalitions while seeking to co-opt civil society groups, (2) harnessing or seeking access to WHO through formal and informal routes and (3) strategically disseminating favourable information.
- There are similarities with the tobacco industry’s behaviour in opposition to WHO’s efforts to advance tobacco control, although much less data are available for the food industry to date.

**What do the new findings imply?**

- Better safeguards against commercial interference and conflicts of interest at the global level as well as the national level have the potential to support WHO’s leading role in action towards better diets.

### Science/Policy intermediaries

Alongside business associations which *overtly* represent business interests, the UPFI has also, more covertly, attempted to influence policy through intermediary organisations or individuals at the intersection of science and policy (science/policy intermediaries (SPIs)) with significant corporate involvement or funding. SPIs include individual scientists,^17 78^ but predominantly involve organisations such as the International Life Sciences Institute (ILSI) (CS-2),^79 80^ the now defunct Global Energy Balance Network (ex-IO-1),^80^ or the International Food Information Council.^79^ In-depth studies of ILSI, for example, conclude that the organisation has promoted industry interests across national and global settings, while enjoying privileged access as a seemingly independent organisation.^79 80^ US Internal Revenue Service filings suggest that ILSI also sponsored WHO internships in 2012, 2013 and 2015.^81–83^ SPIs often facilitate *information management* (see *information management* section below).

Philanthropic institutions such as the Bill and Melinda Gates Foundation, among the top funders of WHO, have significant agenda-setting power in global health and can influence priorities through earmarked contributions.^84^ Four interviewees expressed concerns about the Gates Foundation’s pro-industry stance (IO-3, ex-IO-1, CS-7, CS-8). The most recent tax return of the Bill and Melinda Gates Foundation Trust suggests that it does, for instance, have investments in a number of UPFI corporations, including PepsiCo, Coca-Cola (FEMSA and European Partners), and McDonald’s,^85^ and has funded projects in partnership with the latter.^84^ Such relationships have been perceived as channels for influence by industry allies^17^: ILSI’s founder responded to a US official’s suggestion to lobby WHO through ‘*Gates or Bloomberg people’* with the following: *‘I like the one especially about having Mr. Bill Gates help. Our Chairman knows him well’*.^78^

### Co-opting civil society

Our analysis indicates that the UPFI has consistently attempted to form a closer relationship with civil society in the global NCD space. A number of global NGOs have received funding from the UPFI (CS-1, CS-2, CS-9): the World Heart Federation, for instance, has historically received funds from Unilever^74^ and the International Diabetes Federation from Nestlé.^86 87^ Civil society interviewees reported that UPFI offers of financial support, as well as invites to attend and present at industry-organised events or join industry panels, were common (ex-IO-1, CS-1). One senior advocate recalled:

*I was almost stalked by [soda company] and [soda company]*. *They would have given me as much money as I wanted. You know, I would go to meetings and then suddenly I would find that they were there, and I was invited to go speak to their board*. (CS-1)

They attributed such efforts to the UPFI’s desire to foster credibility and dampen civil society criticism (CS-1, CS-2).

Establishing collaborations with NGOs may also facilitate access to global policymaking. This was illustrated by PepsiCo and Coca-Cola’s active participation in the NCD Roundtable, which was convened to discuss policy recommendations for WHO’s work on NCDs and the 2011 HLM by the Global Health Council, a US-based membership organisation in official relations with WHO.^88^ On becoming members of the Council, PepsiCo and Coca-Cola were able to feed into these policy recommendations via the Roundtable, and qualified to participate in the World Health Assembly and other high-level events as part of its delegation.^89^ Harnessing another privilege granted to the Global Health Council with official relations status, PepsiCo co-sponsored a multi-stakeholder dialogue at the UN in the lead-up to the HLM, affording the company and its invited representatives access to decision-makers.^89^

Seven interviewees described the relationship between civil society and industry as significantly closer in the nutrition space than the more focused NCD space (IO-3, ex-IO-2, CS-3, CS-5, CS-6, CS-7, CS-8, CS-9). For instance, malnutrition-focused organisations such as the Global Alliance for Improved Nutrition foundation and the Scaling up Nutrition Movement have commonly entered or promoted partnerships with major UPFI actors. Scaling up Nutrition also hosts a Business Network, co-convened by the Global Alliance for Improved Nutrition and the UN World Food Programme, which includes PepsiCo, Mars, and Kellogg’s.^90^

#### Hiring former WHO staff

Food corporations have hired former WHO officials (CS-1). For example, Derek Yach, a former Executive Director for NCDs, was recruited by PepsiCo as Senior Vice President in 2007. While employed at WHO under DG Brundtland, Yach played a role in organising dialogues with industry; in his role at PepsiCo, he later sat on the other side of the table.^86 89 91^ PepsiCo also recruited Yach’s previous superior, Gro Harlem Brundtland, to its Blue Ribbon Advisory Board in 2007.^92^ Similarly, Janet Voûte, previously head of the World Heart Federation, led the development of WHO’s NCD Network immediately before joining Nestlé as Global Head of Public Affairs.^93^ This technique can facilitate access to public health communities through former officials’ networks and credibility (CS-1).

### Involvement and influence in policymaking

All types of data we analysed indicated that UPFI actors are able to access WHO policy processes through formal routes, a technique which is facilitated by *coalition management*. There was also evidence of less a prominent technique: intimidating policymakers.

#### Participating in WHO processes

UPFI actors regularly engaged with WHO on NCD policy through consultations, hearings and meetings (IO-1, IO-2, IO-3, IO-4, ex-IO-1, ex-IO-2, CS-3, CS-4, CS-5, CS-6, CS-7, CS-8, CS-9),^38 74 75^ where they consistently opposed statutory regulation favour of voluntary approaches.^45 94^ In line with the WHO’s approach to industry engagement, this primarily occurs through business associations.^34^ Regular dialogues between the WHO DG, officials and the UPFI, first set up in 2003 by DG Brundtland,^91 95^ occur primarily through IFBA, with corporations attending as members (IO-1, IO-2, IO-4, ex-IO-1, CS-6).^96^ At the civil society hearing preceding the third HLM, the speaker panel included IFBA’s Secretary-General.^97^

The ability of individual corporations to engage as members of associations is notable. Nestlé and Unilever, for instance, spoke as IFBA members at a meeting organised by the WHO Global Coordination Mechanism on NCDs.^98 99^ Although business associations are eligible to obtain WHO official relations status, no key UPFI groups currently hold it, after International Special Dietary Industries were stripped of it in 2013^100^ and ILSI in 2015,^79 101 102^ following the revelation that one of its member companies was owned by a tobacco conglomerate. Although it seems that IFBA previously attempted to gain official relations status (IO-3) it has not succeeded to date.^86 88^

#### Intimidating policymakers

Three interviewees discussed 2 separate cases, where UPFI representatives exhibited verbally intimidating behaviour towards IO staff (IO-4, ex-IO-1, ex-IO-2). These interviews are the only evidence we identified of such conduct.

### Information management

Our analysis suggests that the UPFI engaged in information management, simultaneously producing and disseminating information supporting its preferred policy positions and challenging unfavourable information perceived as threatening.

#### Sponsoring or disseminating favourable information

UPFI actors have funded and disseminated research to favourably influence policy debates (CS-1, CS-2, CS-8, ex-IO-1).^79 80^ Industry-linked SPIs in particular play an important role in funding and disseminating favourable research, reports or policy documents. Scientists funded by ILSI, for instance, have supported and enabled the dissemination of industry-favourable narratives.^79 80^ The World Sugar Research Organization commissioned a report warning that the 2004 Global Strategy recommendation on sugar reduction would have severe economic impacts on low-income and middle-income countries,^56^ which was sent to the Food and Agriculture Organization’s DG and national policymakers.^59^ Similarly, one interviewee noted:

*[Business associations] prepare for the [WHO] governing bodies meetings. They design the lobby strategy in terms of policy recommendations they have, and they fund all these think tanks, they prepare policy papers that are leafleted to governments, to missions. And in this, they are much smarter than civil society organisations, I must say*. (CS-8)

Furthermore, IFBA affiliates have published in reputable public health journals, advocating an important role for the UPFI in global health.^91 103 104^ More generally, consultations and hearings provide a platform for the UPFI to advance favourable evidence.^98 99^

#### Challenging or undermining unfavourable information

Simultaneously, UPFI actors have attempted to delegitimise WHO guidance, primarily by denying its evidence base (IO-1, CS-5).^56 58–60 74 79 80^ For example, the sugar industry campaign against the 2004 Global Strategy was rooted in challenges to the science underpinning it.^56 59 72^ Commercial actors may also attempt to suppress unfavourable voices: a study of internal ILSI documents suggest that the organisation ‘*can be deployed to marginalise unfavourable positions, which supports the argument that it is a front for industry when positions need to be quashed’*.^80^ This technique is connected to *civil society co-optation* which may similarly curtail criticism.

#### UPFI reputation management

The UPFI has used corporate social responsibility (CSR) activities, including participation in global public-private partnerships, to maintain and enhance legitimacy in the global health policy space (IO-1, IO-2, CS-6, CS-7).^89 105–107^ Portrayals of corporations as socially responsible are omnipresent in UPFI reports and often invoke the Sustainable Development Goals (SDGs).^108–110^ Alongside partnerships with NGOs, CSR has included contributions to and collaboration with IOs such as the UN Development Programme around access to clean water, and UN Women, supporting female entrepreneurs.^107^ Another example is *Project Last Mile,* which uses Coca-Cola’s supply chain expertise to deliver medicines alongside partners such as the Global Fund and the Bill and Melinda Gates Foundation.^111^ One interviewee noted:

*[…] I can totally understand what’s in it for the Global Fund, having a partnership with Coke. Because Coke, as far as I can see, have the best supply chain in the world. They can get to places that have civil wars. You can’t get rice, but you can get a Coke. […] I don’t think they’ve really reflected on how the SDGs are supposed to be seen as a whole, so you shouldn’t really be partnering with organisations that are detrimental across a whole swathe of other SDGs*. (CS-6)

Despite potentially positive elements, such initiatives may detract from a company’s negative health impacts and serve to *‘blue-wash’* its image through association with IOs.^107^ Moreover, existing partnerships, voluntary initiatives, and commitments around NCDs are consistently promoted as alternatives to statutory regulation.^45^ Two interviewees argued that in its commitments to WHO, the UPFI has predominantly embraced *‘low-hanging fruit’* which poses a lesser threat to profitability, such as trans-fat reduction,^112^ to appear responsible while delaying regulatory action in areas such as sugar reduction and advertising (CS-5, CS-7).

### Perceived differences with tobacco industry behaviour

Interviewees widely described the UPFI as an accepted actor in NCD policy, particularly compared with the tobacco industry. They attributed this primarily to perceived differences in product *‘harmfulness’* and the relative heterogeneity of the food industry (IO-1, ex-IO-2, CS-1, CS-7). Overall, the political behaviour of the UPFI was perceived as less antagonistic than that of the tobacco industry, with two potential explanations given: the comparatively lower degree of regulatory pressure in the dietary NCD policy space and a degree of pre-emption based on the tobacco company misconduct which, when publicised, contributed to the denormalisation of their industry (CS-6, CS-7).

## Discussion

**Figure 1:**
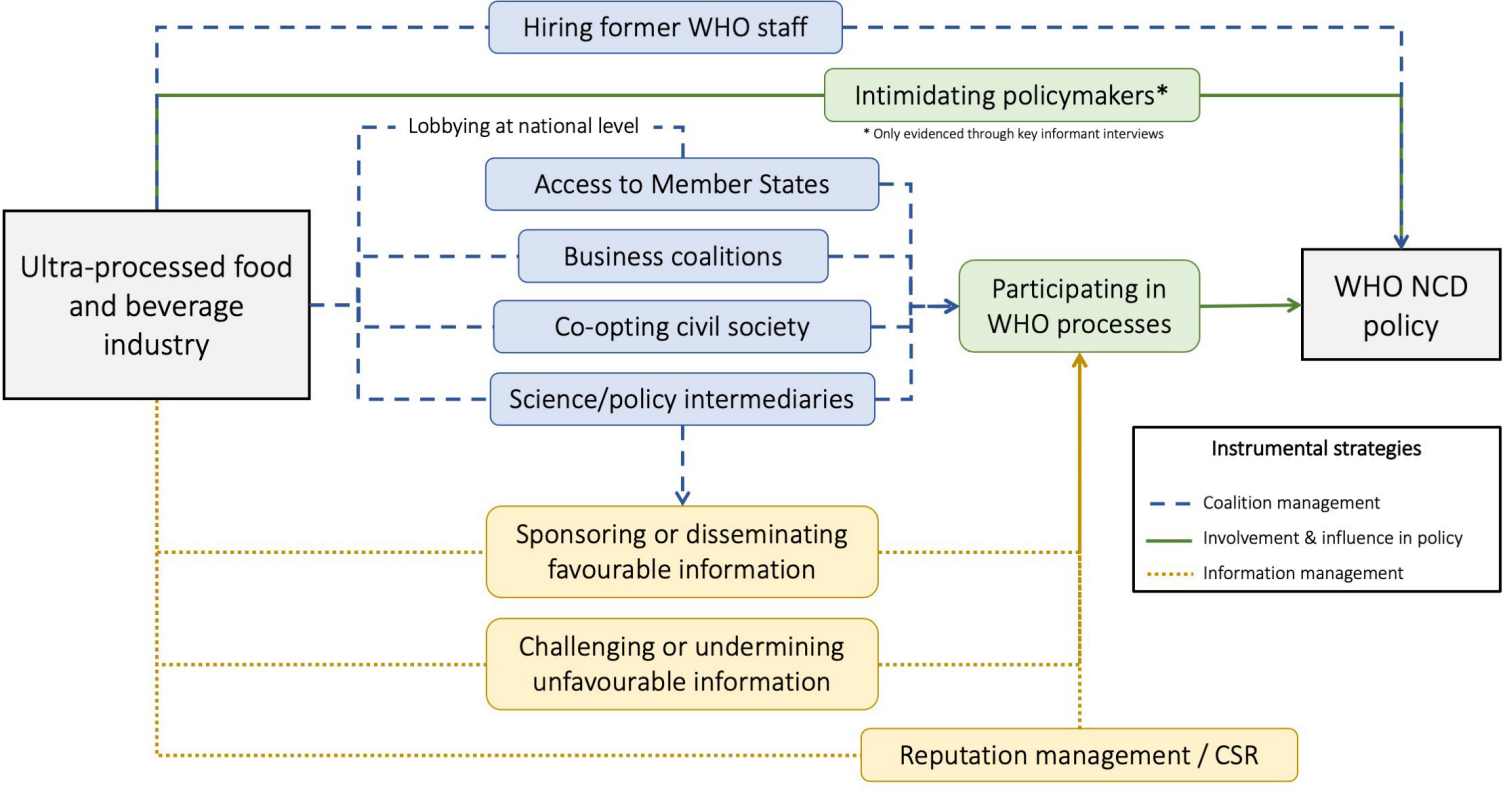
Overview of strategies and techniques identified. CSR, corporate social responsibility; NCD, non-communicable disease.

This study addresses a significant gap in the understanding of UPFI political activity by examining its instrumental strategies in global-level NCD policy. In summary, our analysis suggests that the UPFI uses three overarching strategies which interlink and reinforce each other: coalition management, involvement and influence in policymaking, and information management (figure 1). UPFI actors established and used favourable coalitions—with allied Member States, SPIs, and between businesses—to promote their preferred policy positions, while they sought to co-opt civil society coalitions and hired former WHO staff. Pursuant to WHO guidelines, participation in consultations and meetings primarily takes place through the aforementioned groups. Moreover, the UPFI managed information strategically, funding and promoting evidence and other information favourable to their policy preferences, while challenging unfavourable information. Lastly, UPFI actors’ CSR activities and much-publicised partnerships were attributed to attempts to create a reputation as responsible, legitimate actors.

The first key limitation of this work is that we had restricted access to interviewees, notably those from IOs. This is likely a consequence of the unavoidable limitations imposed by the COVID-19 pandemic as well as the professional risk involved in discussing politically sensitive topics in the absence of freedom of information provisions within the UN system. Second, the documents analysed in this study were drawn from a limited pool of internal industry files. As such, our data do not provide a comprehensive picture of UPFI activities. Third, over half of the identified academic articles were commentaries, news pieces or editorials by individuals involved in WHO processes, rather than original research, which highlights the need for more empirical work in this area. Moreover, WHO is not the only IO involved in dietary NCD policy, thus further research addressing the wider UN system is necessary to better understand the UPFI’s role.

We sought to overcome these limitations in a number of ways. Notably, we used a conceptual model based on high evidential standards from the tobacco literature to structure our enquiry. The PDM has been successfully used to analyse political activities of the UPFI and other industries,^13 14 42–44^ showing similar tactics at the national level and thus providing a priori evidence to suggest comparable tactics at the global level. Nonetheless, there remains a need for a joint-up framework based on rigorous analysis of evidence from multiple unhealthy commodity industries, which would offer a more appropriate basis for cross-industry research. We also triangulated multiple data sources to overcome weaknesses of each.^49^ While we did not approach UPFI representatives for this study, two studies have analysed their public views on NCD policy at the WHO and the industry’s role within it in depth,^45 113^ finding that UPFI groups have consistently opposed statutory regulation in favour of self-regulatory approaches, and promoted a strong role for industry in policymaking while emphasising the limits of WHO’s mandate and fostering a narrow understanding of conflict of interest.^45 113^ Together with the current findings that the UPFI engages in a broad range of techniques to achieve these policy preferences, this renders questionable whether they can meaningfully contribute to policy development as they claim.

The UPFI remains able to exert significant structural, instrumental and discursive power in global health and nutrition governance.^114^ Our analysis accentuates a power imbalance which compromises the ability of global health governance to deliver in the public interest. The food industry is enormously heterogenous, but only a narrow subset of actors has the resources and capacity to engage with IOs. This imbalance means that it is easier for UPFI multinationals to have their voices heard than for parts of the food industry whose practices may be less damaging or even beneficial to health and who may therefore have a role to play, and for civil society or people living with NCDs who are more likely to represent the public interest. Although smaller actors within the food industry are likely to lack the resources for sustainable IO engagement, movements such as La Via Campesina, which has a long history of representing small-scale food producers at the UN Food and Agriculture Organization,^115^ provide a potential route for engagement. Speaking to a further power imbalance, our research highlights how high-income countries hosting major food companies can stymie efforts, primarily of low-income and middle-income countries, to effectively address NCDs. Addressing rather than reinforcing such power imbalances should be central to considerations on engagement and partnerships, at national and global level.

The food industry enjoys a high degree of acceptability in global health policy circles. The heterogeneity of this unhelpfully broad category seems to play a key role in legitimising those companies whose practices are most detrimental to health, and it closely links to UPFI multinationals’ own framing of themselves as *‘part of the solution’* and attempts to mitigate conflict of interest as unfair vilification.^13 45 113 116 117^ However, their political behaviour is strikingly similar to that of the tobacco industry. In fact, our study is one among a growing body of literature which documents parallels between a range of unhealthy commodity industries.^118–120^ Like at national level,^121^ we identify similarities in the political behaviour of the UPFI and tobacco industries at the global level. Analyses of internal documents show the tobacco industry’s aggressive campaign to delay and weaken the WHO FCTC, including lobbying through allied Member States and SPIs to oppose the regulation of their products, and recruiting ex-WHO staff,^46–48 122^ all of which we document the UPFI using here.

Overall, this coherence in behaviour demands greater coherence in governance approaches to unhealthy commodity industries.^33 123 124^ This is likely to remain contentious in light of the public health community’s ambiguity on interactions with the food industry compared with other unhealthy commodity industries,^125^ but others have suggested that the FCTC, specifically the way in which it deals with the conflict of interest between the tobacco industry and public health, provides a potential pathway for action on healthier diets.^33^ Although arguably neither perfect nor easy to implement at country level, FCTC Article 5.3, which aims to protect public health policymaking from tobacco industry interference, has had some success in reinforcing tobacco control.^126–128^ Our findings suggest that comprehensive, effective safeguards against undue influence and conflicts of interest are paramount for WHO to fulfil its role as a global leader in tackling NCDs. This is especially pertinent in light of the new WHO Foundation which renders it possible to circumvent WHO rules on corporate donations and thus poses a clear threat to the agency’s independence,^124^ as illustrated by a widely publicised donation from Nestlé in April 2021.^129^

The need for comprehensive safeguards against commercial interference inevitably extends to national settings: UPFI attempts to influence NCD policy through Member State governments have the potential to impede urgently needed action to address dietary NCDs globally. While we highlight such activities at the global level, there is evidence that similar mechanisms are at work between countries: some of our interviewees reported joint lobbying by the Italian embassy and a food company in Latin American countries, and after Chile introduced ground-breaking marketing restrictions and labelling rules, a public health official reported being accused of ‘*food terrorism’* by a Ferrero executive and the Italian ambassador.^130^

We also observe tensions between attempts to address NCDs on one hand and undernutrition and micronutrient deficiencies on the other hand, the latter often affording a substantial role to the UPFI.^131^ With the double burden of malnutrition and NCDs prevalent within countries, communities and even individuals,^132^ undernutrition and overnutrition cannot be tackled separately, particularly considering that the distribution of food products by multinational corporations, a key driver of unhealthy diets, is touted as a solution to the latter by some. This underlines the need to adopt a focus on double-duty actions which tackle the common drivers of malnutrition.^133^

## Conclusions

This work provides a means of understanding and thus addressing how the UPFI attempts to prevent or weaken regulation at global level. COVID-19 has reminded us that a strong WHO is essential for global public health. What happens in global-level policymaking, and what does not, is important. Strong recommendations on NCDs from the UN and its agencies can provide Member States with a mandate to act, while their absence offers the UPFI a means to oppose regulation. Reconsidering multistakeholder approaches which have allowed commercial interests a seat at the public health policy table may be key to rebuilding a healthier world post-COVID-19.^134^ By highlighting UPFI attempts to interfere with WHO’s efforts to curb NCDs, we provide a foundation for further steps to protect the agency’s work against a set of problems that presents an even greater long-term challenge to global health than the current pandemic. The implementation of stronger conflict of interest measures would be an important first step in addressing power imbalances between Member States, the UPFI, and civil society to more effectively address dietary NCDs.

## Data Availability

All documentary data and literature relevant to the study are publicly available. Interview data will not be shared to maintain confidentiality.
