# Supplementary material for: Big food and the World Health Organization: a qualitative study of industry attempts to influence global-level non-communicable disease policy
Source: BMJ Glob Health. 2021 Jun 11;6(6):e005216. doi: 10.1136/bmjgh-2021-005216 (PMC8202098; doi:10.1136/bmjgh-2021-005216)
Supplement: Supplementary data [file bmjgh-2021-005216supp001.pdf]

Supplementary file 1: Search strategy

SF1 Table. Search terms used to identify relevant academic articles. Last updated 2 October 2020.

Database	Search type & limitations	Search terms	Results
**Web of Science**	Topic (title, abstract, keywords); 2000 and after	((corporat* OR industry OR commercial OR compan*) NEAR/5 ("political activity" OR influenc* OR interfere* OR lobb* OR involve*)) AND (food OR beverage OR sugar) AND ("world health organization" OR "united nations")	49
**PubMed**	Title/abstract; 2000 and after	(corporat* OR industry OR commercial OR compan*) AND ("political activity" OR influenc* OR interfere* OR lobb* OR involve*) AND (food OR beverage OR sugar) AND ("world health organization" OR "united nations")	59
**Scopus**	Title, abstract, keywords; 2000 and after	(corporat* OR industry OR commercial OR compan*) AND ("political activity" OR influenc* OR interference OR lobb* OR involvement) AND (food OR beverage OR sugar) AND ("world health organization" OR "united nations")	192
**Google Scholar**	n/a	(corporate OR industry OR commercial OR company) + ("political activity" OR influence OR interference OR lobby OR involvement) + (food OR beverage OR sugar) + ("world health organization" OR "united nations") + "global health"	17,400 (first 25 pages of results screened)
