# Supplementary material for: Big food and the World Health Organization: a qualitative study of industry attempts to influence global-level non-communicable disease policy
Source: BMJ Glob Health. 2021 Jun 11;6(6):e005216. doi: 10.1136/bmjgh-2021-005216 (PMC8202098; doi:10.1136/bmjgh-2021-005216)
Supplement: Supplementary data [file bmjgh-2021-005216supp002.pdf]

Supplementary file 2: Included literature and internal industry documents

SF2 Table 1. Included academic literature from systematic searches.

Title	Journal	Author(s)	Year	Type (classification from publisher)
Political context of the World Health Organization: sugar industry threatens to scupper the WHO	*Int J Health Serv*	Boseley, S.	2003	Research Article
Why the Bush administration and the global sugar industry are determined to demolish the 2004 WHO global strategy on diet, physical activity and health	*Public Health Nutrition*	Cannon, G.	2004	Research
Addressing NCDs: Penetration of the Producers of Hazardous Products into Global Health Environment Requires a Strong Response: Comment on "Addressing NCDs: Challenges From Industry Market Promotion and Interferences"	*International journal of health policy and management*	Casswell, S.	2019	Commentary
Will industry influence derail UN summit?	*British Medical Journal*	Cohen, D.	2011	Feature
US government rejects WHO's attempts to improve diet	*British Medical Journal*	Dyer, O.	2004	News
United States wins more time to lobby against WHO diet plan	*British Medical Journal*	Dyer, O.	2004	News
The palm oil industry and noncommunicable diseases	*Bulletin of the World Health Organization*	Kadandale, S., Marten, R., Smith, R.	2018	Research
Transnational corporations and oral health: examples from the sugar industry	*Community Dent Health*	Kearns, C. E., Watt, R. G.	2019	Research
Non-communicable disease governance in the era of the sustainable development goals: a qualitative analysis of food industry framing in WHO consultations	*Globalization and Health*	Lauber, K., Ralston, R., Mialon, M., Carriedo, A., Gilmore, A. B.	2020	Research
Public Meets Private: Conversations Between Coca-Cola and the CDC	*The Milbank Quarterly*	Maani, N., Ruskin, G., McKee, M., Stuckler, D.	2019	Research
Take me to your liter: politics, power and public-private partnerships with the sugar-sweetened beverage industry in the post-2015 development agenda.	Washington International Law Journal	Moscetti, C. W., Taylor, A. L.	2015	Research

World Health Organization's Global Strategy on diet, physical activity and health: the process behind the scenes	*Scandinavian Journal of Nutrition*	Norum, K. R.	2005	Not specified
Philanthrolateralism: Private Funding and Corporate Influence in the United Nations	*Global Policy*	Seitz, K., Martens, J.	2017	Special Issue Article
Are industry-funded charities promoting "advocacy-led studies" or "evidence-based science"?: a case study of the International Life Sciences Institute	*Globalization and Health*	Steele, S., Ruskin, G., Sarcevic, L., McKee, M., Stuckler, D.	2019	Research
Pushing partnerships: corporate influence on research and policy via the International Life Sciences Institute	*Public Health Nutrition*	Steele, S., Ruskin, G., Stuckler, D.	2020	Research
Commentary: UN high level meeting on non-communicable diseases: an opportunity for whom?	*British Medical Journal*	Stuckler, D., Basu, S., McKee, M.	2011	Commentary
Global Health Philanthropy and Institutional Relationships: How Should Conflicts of Interest Be Addressed?	*PLOS Medicine*	Stuckler, D., Basu, S., McKee, M.	2011	Policy Forum
Textual analysis of sugar industry influence on the World Health Organization's 2015 sugars intake guideline	*Bulletin of the World Health Organization*	Stuckler, D., Reeves, A., Loopstra, R.	2016	Research
The WHO Global Strategy on Diet, Physical Activity and Health: The controversy on sugar	*Development*	Waxman, A.	2004	Thematic Section
Politics of International Health in the Bush Administration	*Development*	Waxman, H. A.	2004	Thematic Section
Commercial influence in control of non-communicable diseases	*British Medical Journal*	Whitaker, K., Webb, D., Linou, N.	2018	Editorial

SF2 Table 2. Included internal food industry documents from searches of the Food Industry Documents Library.

Title	Author(s)	Year	Link
Letter to Tommy G Thompson from American Advertising Foundation Regarding Draft Report of the Joint WHO and FAO Expert Consultation on Diet, Nutrition and the Prevention of Chronic Diseases	American Advertising Federation AAoAA, American Bakers Association; American Feed Industry Association, American Frozen Foods Institute, American Meat Institute, Animal Health Institute, Biotechnology Industry Organization, Consumer Health Care Products Association, Council for Responsible Nutrition, Frozen Potato Products Institute, Grocery Manufacturers of America, International Dairy Foods Association, International Council of Grocery Manufacturers Associations, National Confectioners Association; Chocolate Manufacturers Association, National Council of Chain Restaurants, National Grocers Association, National Restaurant Association, National Soft Drink Association, Snack Food Association, Wheat Foods Council, US Chamber of Commerce, US Council for International Business	2002	https://www.industrydocuments.ucsf.edu/docs/mtck0229
Management by Objectives	Unknown	2003	https://www.industrydocuments.ucsf.edu/docs/gtpj0226
Performance and Compensation Review and Consideration	Briscoe, A.	2004	https://www.industrydocuments.ucsf.edu/docs/xtpj0226
Achievement for Discussion During Executive Session	Briscoe, A.	2004	https://www.industrydocuments.ucsf.edu/docs/htpj0226
ILSI draft IRS Form 990 for 2013	Brueggemeyer, B.	2014	https://www.industrydocuments.ucsf.edu/docs/yqkk0228
International Government Relations & Public Affairs - Activities Week of March 16, 2015	Cherry, K.	2015	https://www.industrydocuments.ucsf.edu/docs/fmdl0226
FW: INFORM: International Food & Beverage Alliance 2014 Progress Report	Goltzman, M.	2015	https://www.industrydocuments.ucsf.edu/docs/hzcl0226
FW: UPDATED INFORM: World	Goltzman, M.	2015	https://www.industrydocuments.ucsf.edu/docs/kjdl0226

Health Assembly - May 26			
Re: Daily European Ness Flash - 25.06.15	Malaspina, A.	2015	https://www.industrydocuments.ucsf.edu/docs/kpcy0227
[Email from Alex Malaspina concerning the Daily European News Flash]	Peter, J. C., Malaspina, A.	2015	https://www.industrydocuments.ucsf.edu/docs/qxyk0228
Reminder for the Mid-Year ILSI Board of Trustees Conference Call -- Thursday, July 9, 2015	Harris, S.	2015	https://www.industrydocuments.ucsf.edu/docs/pjlk0228
ILSI's Official Relations with the World Health Organization Discontinued	Hentges, E.	2015	https://www.industrydocuments.ucsf.edu/docs/kzkk0228
INFORM: Israel tax threat	Goltzman, M., Banks, H.	2016	https://www.industrydocuments.ucsf.edu/docs/tmcl0226
INFORM: WHO Europe Report on Adolescent Health calls softdrinks "a concern"	Goltzman, M., Vermeulen, W,	2016	https://www.industrydocuments.ucsf.edu/docs/tncl0226
[INFORM: 3-4 May BIAC Forum, Industry Organized OECD on Innovation in Health and Well-Being]	Goltzman, M., Vermeulen, W.	2016	https://www.industrydocuments.ucsf.edu/docs/tjcl0226
FW: INFORM: World Health Assembly May 23-28	Goltzman, M., Vermeulen, W.	2016	https://www.industrydocuments.ucsf.edu/docs/kqcl0226
